# Progression to Diabetes Among Older Adults With Hemoglobin A_1c_–Defined Prediabetes in the US

**DOI:** 10.1001/jamanetworkopen.2022.8158

**Published:** 2022-04-20

**Authors:** Alain K. Koyama, Kai McKeever Bullard, Meda E. Pavkov, Joohyun Park, Russ Mardon, Ping Zhang

**Affiliations:** 1 Division of Diabetes Translation, Centers for Disease Control and Prevention, Atlanta, Georgia; 2 Westat, Rockville, Maryland

## Abstract

This cohort study estimates the progression to diabetes among older adults with hemoglobin A_1c_–defined prediabetes in clinical settings in the US.

## Introduction

Data on progression to diabetes in older adults are essential to guide policy because lifestyle intervention programs may not be cost-effective when progression rates are low.^1^ Existing data are limited and show a wide range of progression rates.^2,3,4^ Although recommendations for using hemoglobin A_1c_ (HbA_1c_) to diagnose prediabetes are common, the progression rate of HbA_1c_-defined prediabetes in clinical settings among older US adults remains unclear. We estimated the annual progression rate (APR) of HbA_1c_-defined prediabetes in adults 65 years or older from the Longitudinal Epidemiologic Assessment of Diabetes Risk (LEADR) study.^5^

## Methods

The LEADR study comprises longitudinal outpatient electronic health record (EHR) data on 2 045 999 adults between January 2010 and December 2018 across 10 geographically diverse US health care networks.^5^ Analyses were completed in October 2021. The Centers for Disease Control and Prevention institutional review board deemed the study exempt from review, with a waiver of informed consent because LEADR data are deidentified. Incident diabetes was ascertained using previously published criteria.^6^ This cohort study included patients 65 years or older with HbA_1c_ level of 5.7% to 6.4% (ie, prediabetes), with 3 months or more follow-up after HbA_1c_-based prediabetes diagnosis, and without kidney failure. The APR was estimated from mean 1-year cumulative incidence (eg, [cases/patients]/mean follow-up years), and 95% CIs were derived from Poisson regression models. Estimates were stratified by baseline variables: age group, sex, race and ethnicity, social vulnerability index (SVI), body mass index (BMI), HbA_1c_ level, family history of diabetes, and hypertension diagnosis. Analyses were performed using SAS, version 9.4. This study followed the STROBE reporting guideline.

## Results

A total of 50 152 patients were included in the study (Table 1). Median follow-up was 2.3 years (IQR, 1.2-3.7 years). Crude diabetes incidence was 53 per 1000 person-years (APR, 5.3%; 95% CI, 5.1%-5.4%) (Table 2). APRs were 5.0% or greater for all groups except patients with the lowest SVI, BMI less than 30, or baseline HbA_1c_ level of 5.7% to 5.9%. The most pronounced differences in progression were for BMI and HbA_1c_. The APR among patients with BMI of 18.5 to 24.9 was 3.5% (95% CI, 3.3%-3.7%), whereas it was 7.6% (95% CI, 7.0%-8.3%) among those with BMI of 40 or greater. Patients with HbA_1c_ levels of 5.7% to 5.9% had an APR of 2.8% (95% CI, 2.7%-2.9%) compared with 8.2% (95% CI, 7.9%-8.4%) among patients with HbA_1c_ levels of 6.0% to 6.4%.

**Table 1.** Baseline Characteristics of Study Patients With Prediabetes^a^

Characteristic	Patients, No. (%) (N = 50 152)
Age, y	
65-69	18 075 (36.0)
70-74	13 088 (26.1)
75-79	9210 (18.4)
≥80	9779 (19.5)
Sex	
Female	29 372 (58.6)
Male	20 780 (41.4)
Race and ethnicity^b^	
Asian/Pacific Islander	1408 (2.8)
Hispanic	5537 (11.0)
Non-Hispanic Black	4857 (9.7)
Non-Hispanic White	36 361 (72.5)
Other^c^	638 (1.3)
Unknown	1351 (2.7)
Social vulnerability index score^d^	
0-0.24	11 607 (23.1)
0.25-0.49	14 906 (29.7)
0.50-0.74	10 151 (20.2)
0.75-1.00	6843 (13.6)
Missing	6645 (13.3)
Body mass index^e^	
<18.5	789 (1.6)
18.5-24.9	11 496 (22.9)
25.0-29.9	18 173 (36.2)
30.0-34.9	11 624 (23.2)
35.0-39.9	4733 (9.4)
≥40.0	2566 (5.1)
Missing	771 (1.5)
HbA_1c_ level, %	
5.7-5.9	27 204 (54.2)
6.0-6.4	22 948 (45.8)
Family history of diabetes^f^	2929 (5.8)
Hypertension diagnosis	29 918 (59.7)

Abbreviation: HbA_1c_, hemoglobin A_1c_.

SI conversion factor: To convert percentage of total HbA_1c_ to proportion of total HbA_1c_, multiply by 0.01; to convert glucose to mmol/L, multiply by 0.0555.

^a^ Prediabetes was defined as baseline HbA_1c_ level of 5.7% to 6.4%.

^b^ Self-reported race and ethnicity are reported because they are socioeconomic factors that may be associated with progression to diabetes.

^c^ Other data were from the electronic health record and included Alaska Native, American Indian, American Indian or Alaska Native, multiple race, other, or other race.

^d^ Derived from US census measures of the patient’s zip code and comprises socioeconomic status, household composition and persons with disability, racial and ethnic minority group and language, and housing and transportation. The overall score represents a sum of the 4 categories, with higher values indicating greater social vulnerability.

^e^ Calculated as weight in kilograms divided by height in meters squared.

^f^ Cases documented in the electronic health record. The Longitudinal Epidemiologic Assessment of Diabetes Risk data set excludes patients with prevalent diabetes at their first encounter or in the following 12 months based on a diabetes diagnosis, prescription for an antidiabetic agent, or laboratory results (HbA_1c_ level ≥6.5%, fasting plasma glucose level ≥126 mg/dL, or random blood glucose level ≥200 mg/dL).

**Table 2.** Diabetes Progression Rate Among 50 152 Patients With Prediabetes by Sociodemographic and Clinical Characteristics^a^

Characteristic	Diabetes cases, No.	Person-years, No.	Annual progression rate, % (95% CI)
Overall	7161	136 058	5.3 (5.1-5.4)
Age, y			
65-69	2735	51 047	5.4 (5.2-5.6)
70-74	1946	35 505	5.5 (5.2-5.7)
75-79	1290	25 698	5.0 (4.8-5.3)
≥80	1190	23 807	5.0 (4.7-5.3)
Sex			
Female	4029	80 629	5.0 (4.8-5.2)
Male	3132	55 429	5.7 (5.5-5.9)
Race and ethnicity^b^			
Asian/Pacific Islander	246	4293	5.7 (5.1-6.5)
Hispanic	956	17 977	5.3 (5.0-5.7)
Non-Hispanic Black	841	14 427	5.8 (5.5-6.2)
Non-Hispanic White	4848	94 837	5.1 (5.0-5.3)
Other^c^	94	1497	6.3 (5.1-7.7)
Unknown	176	3026	5.8 (5.0-6.7)
Social vulnerability index score^d^			
0-0.24	1408	32 408	4.3 (4.1-4.6)
0.25-0.49	2149	42 537	5.1 (4.8-5.3)
0.50-0.74	1786	28 287	6.3 (6.0-6.6)
0.75-1.00	1209	20 502	5.9 (5.6-6.2)
Missing	609	12 324	4.9 (4.6-5.4)
Body mass index^e^			
<18.5	77	1964	3.9 (3.1-4.9)
18.5-24.9	1105	31 862	3.5 (3.3-3.7)
25-29.9	2416	49 768	4.9 (4.7-5.1)
30-34.9	2009	31 231	6.4 (6.2-6.7)
35-39.9	922	12 598	7.3 (6.9-7.8)
≥40	510	6699	7.6 (7.0-8.3)
Missing	122	1936	6.3 (5.3-7.5)
HbA_1c_ level, %			
5.7-5.9	2078	73 729	2.8 (2.7-2.9)
6.0-6.4	5083	62 328	8.2 (7.9-8.4)
Family history of diabetes^f^			
No	6748	130 117	5.2 (5.1-5.3)
Yes	413	5940	7.0 (6.3-7.7)
Hypertension diagnosis			
No	3106	61 596	5.0 (4.9-5.2)
Yes	4055	74 462	5.4 (5.3-5.6)

Abbreviation: HbA_1c_, hemoglobin A_1c_.

SI conversion factor: To convert percentage of total HbA_1c_ to proportion of total HbA_1c_, multiply by 0.01; to convert glucose to mmol/L, multiply by 0.0555.

^a^ Incident diabetes required a minimum of 2 records of diabetes diagnoses, diabetes drug prescriptions, and/or laboratory measurements (HbA_1c_ level ≥6.5%, fasting blood glucose level ≥126 mg/dL, or random blood glucose level ≥200 mg/dL) occurring within 2 years of each other. Events had to occur on separate days, and prescriptions for metformin, thiazolidinediones, and glucagon-like peptide 1 agonists had to be combined with another type to count.

^b^ Self-reported race and ethnicity are reported because they are socioeconomic factors that may be associated with progression to diabetes.

^c^ Other data were from the electronic health record and included Alaska Native, American Indian, American Indian or Alaska Native, multiple race, other, or other race.

^d^ Derived from US census measures of the patient’s zip code and comprises socioeconomic status, household composition and persons with disability, racial and ethnic minority group and language, and housing and transportation. The overall score represents a sum of the 4 categories, with higher values indicating greater social vulnerability.

^e^ Calculated as weight in kilograms divided by height in meters squared.

^f^ Cases documented in the electronic health record. The Longitudinal Epidemiologic Assessment of Diabetes Risk data set excludes patients with prevalent diabetes at their first encounter or in the following 12 months based on diabetes diagnosis, prescription for an antidiabetic agent, or laboratory results (HbA_1c_ level ≥6.5%, fasting plasma glucose level ≥126 mg/dL, or random blood glucose level ≥200 mg/dL).

## Discussion

The estimated APR to diabetes among older adults with prediabetes in this study was 5.3%, differing from previous US studies, likely owing to different study designs and populations. A study of community-dwelling Black and White adults 70 years or older with HbA_1c_-defined prediabetes reported approximately half the APR in our study and substantial regression to normoglycemia, recommending against prediabetes screening and intervention in older adults.^2^ The results of that study may be attributed to an older, less diverse sample and self-selection bias from an observational design.^2^ Using HbA_1c_ and fasting blood glucose levels to define prediabetes, a study of primary care patients reported an APR of 4.7% among individuals aged 65 to 75 years and 4.1% among those 75 years or older.^3^ Another study of a nationally representative population reported APRs of 4.5% among those aged 65 to 79 years and 1.8% among those 80 years or older, both with HbA_1c_-defined prediabetes.^4^

Strengths of this study include the large sample of diverse patients. Limitations include patients’ unknown duration of prediabetes, possible incomplete capture of health care utilization resulting in under-ascertainment of prediabetes or diabetes, and the inability to distinguish between type 1 and type 2 diabetes. Owing to inherent selection bias, the EHR-based sample was representative of patients comprising the health care organizations contributing data and may not be representative of the general US population. Our findings may provide important information to evaluate the cost-effectiveness of lifestyle interventions in older adults with prediabetes identified by HbA_1c_ testing in clinical settings.

## References

[1] Park J, Zhang P, Shao HUI, Laxy M, Imperatore G. 141-OR: selecting intervention population for lifestyle program for type 2 diabetes prevention (LPT2DP): a cost-effectiveness perspective. Diabetes. 2021;70(suppl 1):141-OR. doi:10.2337/db21-141-OR PMC9578149 35434784

[2] Rooney MR, Rawlings AM, Pankow JS, . Risk of progression to diabetes among older adults with prediabetes. JAMA Intern Med. 2021;181(4):511-519. doi:10.1001/jamainternmed.2020.8774 33555311 PMC7871207

[3] DeJesus RS, Breitkopf CR, Rutten LJ, Jacobson DJ, Wilson PM, Sauver JS. Incidence rate of prediabetes progression to diabetes: modeling an optimum target group for intervention. Popul Health Manag. 2017;20(3):216-223. doi:10.1089/pop.2016.0067 27689627

[4] Bardenheier BH, Wu WC, Zullo AR, Gravenstein S, Gregg EW. Progression to diabetes by baseline glycemic status among middle-aged and older adults in the United States, 2006-2014. Diabetes Res Clin Pract. 2021;174:108726. doi:10.1016/j.diabres.2021.108726 33662490

[5] Fishbein HA, Birch RJ, Mathew SM, . The Longitudinal Epidemiologic Assessment of Diabetes Risk (LEADR): unique 1.4 M patient electronic health record cohort. Healthc (Amst). 2020;8(4):100458. doi:10.1016/j.hjdsi.2020.100458 33011645 PMC11008431

[6] Nichols GA, Desai J, Elston Lafata J, ; SUPREME-DM Study Group. Construction of a multisite DataLink using electronic health records for the identification, surveillance, prevention, and management of diabetes mellitus: the SUPREME-DM project. Prev Chronic Dis. 2012;9:E110. doi:10.5888/pcd9.110311 22677160 PMC3457753

